# A national study of Continuous Professional Competence (CPC) amongst pre-hospital practitioners

**DOI:** 10.1186/s12913-015-1197-1

**Published:** 2015-12-02

**Authors:** Shane Knox, Walter Cullen, Colum P. Dunne

**Affiliations:** Graduate Entry Medical School and Centre for Interventions in Infection, Inflammation & Immunity (4i), University of Limerick, Castletroy, Limerick, Ireland; Health Services Executive, National Ambulance Service College, Dublin, Ireland

**Keywords:** EMTs, Paramedics, Advanced Paramedics, Continuous Professional Development, Continuous Professional Competence

## Abstract

**Background:**

Internationally, continuing professional competence (CPC) is an increasingly important issue for all health professionals. With the introduction of the first CPC framework for Emergency Medical Technicians (EMTs) and the imminent introduction of CPC for Paramedics and Advanced Paramedics (APs) in Ireland, this study aimed to identify attitudes towards CPC and factors that might influence such a framework.

**Methods:**

All EMTs (*n* = 925), Paramedics and APs (*n* = 1816) registered in Ireland were invited by email to complete an anonymous on-line survey. The study instrument was designed based on continuous professional development (CPD) questionnaires used by other healthcare professions. Quantitative and qualitative analyses were performed.

**Results:**

The overall response rates were: EMTs 43 % (*n* = 399), Paramedics and APs 43 % (*n* = 789), with 82 % of APs and 38 % of Paramedics participating. The majority of participants in all groups agreed that registration was of personal importance and that evidence of CPC should be maintained; 39 % of Paramedics/APs and 78 % of EMTs believed that persistent failure to meet CPC requirements should mandate denial of registration. From a pre-determined list of activities, in excess of 88 % of all respondents indicated practical training scenarios, cardiac re-certification, e-learning supplemented by related practice, and training with simulation manikins were most relevant to these roles. However, least relevant to them were: e-learning alone (Paramedic/AP 36 %; EMT 35 %); project work (Paramedic/AP 27 %; EMT 48 %); and appraisal of journal articles (Paramedic/AP 24 %; EMT 39 %).

**Conclusion:**

Irish EMTs, Paramedics and Advanced Paramedics were supportive of CPC and favoured a ‘mixed’ model approach which includes: blended learning, practical skills, simulation, practical/team-based exercises, e-learning combined with practical skills, and evidence of patient contact. It is hoped that these insights will inform the CPC guidelines to be introduced.

**Electronic supplementary material:**

The online version of this article (doi:10.1186/s12913-015-1197-1) contains supplementary material, which is available to authorized users.

## Background

Ambulance services provided by pre-hospital practitioners in Ireland are governed by the Regulator, the Pre-Hospital Emergency Care Council (PHECC), an independent statutory agency responsible for implementing standards of education and training for pre-hospital emergency care practitioners.

The Regulator maintains a register of pre-hospital practitioners and those licensed are permitted legally to practice using guidelines developed by the Regulator to manage patients. There are three level of practitioner: Emergency Medical Technician (EMT), Paramedic and Advanced Paramedic (AP).

At a professional level, pre-hospital care in Ireland is provided by the Health Service Executive’s (HSE) National Ambulance Service (NAS) and (in parts of Dublin city) the ‘Dublin Fire Brigade’. Staff who respond to pre-hospital incidents are all trained to Paramedic or Advanced Paramedic (AP) level. In addition, pre-hospital care is provided at sporting and other public events by Emergency Medical Technicians, mostly affiliated to voluntary organisations: e.g., Civil Defence, Order of Malta Ireland, St. John Ambulance and the Irish Red Cross.

In Ireland, currently, once qualified there is no regulatory requirement for the practitioner, at paramedic or advanced paramedic level, to provide evidence of competence, or any link between competence and registration to practice. However, it is reasonable that practitioners and consumers alike view maintenance of competency as a basic element of ethical and responsible practice [1]. Therefore, the Regulator’s strategic plan (2011–2014) stated the need to develop and implement a continuous professional competence (CPC) framework [2].

One of the functions of a healthcare Regulator is to protect the public by ensuring that acceptable standards of care are being provided [3]. Previous studies have assessed EMT, Paramedic and Advanced Paramedic (AP) training and continuing education in Ireland [4–6] and internationally [7–9]. However, in this study we wished to determine, for the first time, the attitudes of Irish EMTs, Paramedics and APs towards CPC, their preferred activities, delivery formats and perceived relevance to their roles.

It is accepted that any form of compulsory education is incongruent with the nature of both being a professional and adult; professionals should be self-directed sufficiently to participate autonomously in educational activities rather than being compelled to do so [10]. That, combined with a proliferation of training and education formats that, without justification through specific needs assessment, are unlikely to be effective [11] encouraged us to devise a short answer survey to guide and inform the impending CPC implementation in Ireland.

Additionally, such an approach appears to be relatively rare in the published literature and may, therefore, inform or prove useful to others engaged in developing pre-hospital or other professional CPC/CPD or competency standards in other Countries.

## Methods

### Participants

In February 2012, all registered Paramedics and APs in Ireland with valid email addresses (*n* = 1816) were contacted and provided a link to a Survey Monkey™ online study instrument and to a concise, unbiased explanation of the survey topic. Similarly, in July/August 2012, all registered EMTs (*n* = 925) were contacted by email and provided a similar link to the on-line survey. A written request to the Registrar, the person responsible for maintaining the Register of pre-hospital practitioners, was made outlining the purpose of the research and seeking permission to contact these registrants. Written permission was granted on the basis that the Registrar would circulate the survey details on behalf of the authors. A letter of introduction was sent to registrants to explain the purpose of the research and described how consent would be implied should registrants participate in the research. Participation was voluntary and anonymous. The design and conducting of the study, taking into consideration published healthcare professions’ questionnaires relating to continuous professional development (CPD) [12–14] were approved by the Ethics Committee of the Faculty of Education and Health Sciences, University of Limerick, Ireland and the Research Ethics Committee of the Health Services Executive Mid-Western Regional Hospital, Limerick, Ireland.

### Data collection and analysis

Health professionals are increasingly expected to identify their own learning needs through self-assessment [15, 16]. Therefore, the survey questions were designed to elicit participants’ views on CPC. The survey was piloted after a presentation regarding CPC to 120 EMTs at a biannual conference in 2011 [17]. Responses were recorded and summarized at the event using mind mapping software (mindGenius®). Following analysis of the exercise, the design of the questionnaire was finalised and trialed using 12 EMTs and, subsequently, by a further 20 registered Paramedics/APs. All trial participants were excluded from the study analyses.

The questionnaire (Additional File 1) comprised questions relating to: demographics; opinions regarding CPC; CPC portfolio development; linkage of CPC and registration. The response data were downloaded from Survey Monkey™ software to an electronic data file and quantitative analysis was performed using SPSS version 20.0. To make analysis more meaningful, responses to the five-point Likert scale were analysed using three options, ‘strongly agree/agree’, ‘undecided’ and ‘strongly disagree/disagree’. Not every question was answered by every respondent and, therefore, answers are described by number and percentage of responses to specific questions.

## Results

### Demographics

**Fig. 1:**
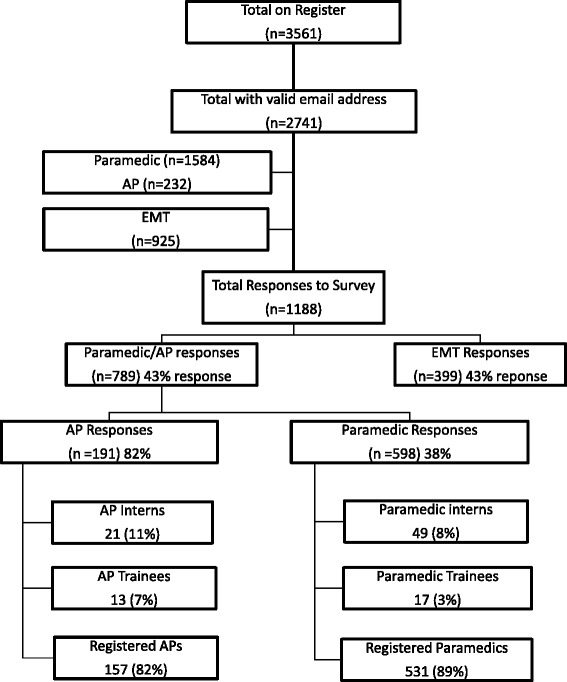
Registered Emergency Medical Technicians (EMTs), Paramedics and Advanced Paramedics (APs) in Ireland and number of responses

**Fig. 2:**
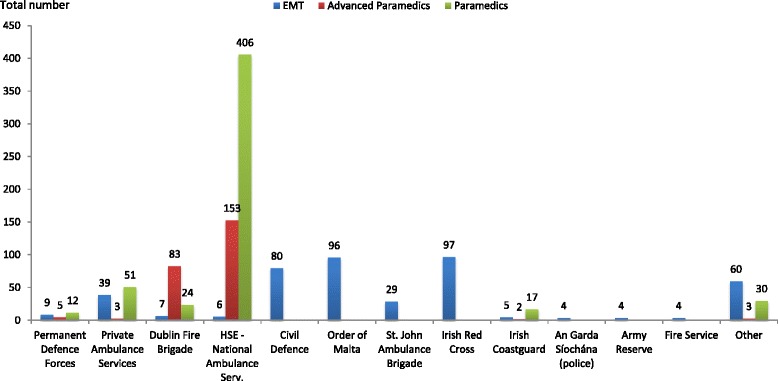
Total amount of responses from Emergency Medical Technicians (EMTs), Paramedics and Advanced Paramedics (APs) based on organisation

There were 789/1816 responses received from Paramedics and Advanced Paramedics (APs), 43 % of all registered Paramedics and APs with email addresses, of whom 598 were Paramedics (38 % of the national cohort) and 191 were Advanced Paramedics (82 % of the national cohort). From the EMT cohort, there were 399/925 responses received (43 % of all registered EMTs) (Fig. 1). The majority of respondents were male: Paramedic/AP 85 %, 670: EMT 70 %, 271; with female responses of 15 % Paramedic/AP and 30 % EMT (Table 1). EMT responses were reasonably well dispersed across the Irish voluntary organisations: Order of Malta (96, 24 %), Civil Defence (80, 20 %), St. John Ambulance (29, 7 %) and the Irish Red Cross (97, 24 %). There was considerably less participation by EMTs employed by the Irish State (10 %) such as the Defence Forces, Irish Health Service, An Garda Síochána (police), Fire Service, Coastguard, etc. and private ambulance services (10 %) with those described as ‘not affiliated’ to an organisation (5 %). Paramedic and AP respondents predominantly served in the Irish National Ambulance Service (71 %) and the Dublin Fire Brigade (14 %) (Fig. 2).

**Table 1:** Gender and registration level

Registration Status with Regulatory Body (PHECC)	Male	Female	Total
Advanced Paramedic	138 (88 %)	19 (12 %)	157
Advanced Paramedic Intern	19 (90 %)	2 (10 %)	21
Advanced Paramedic Trainee	11 (85 %)	2 (15 %)	13
Paramedic	451 (85 %)	81 (15 %)	532
Paramedic Intern	38 (78 %)	11 (22 %)	49
Paramedic Trainee	14 (82 %)	3 (18 %)	17
Emergency Medical Technician (EMT)	271 (70 %)	115 (30 %)	386
Response Totals	**941 (80 %)**	**234 (20 %)**	**1175**
Gender not reported			13
Response Percentage: EMT	70 %	30 %	
Response Percentage: paramedic/advanced paramedic	85 %	15 %

### Attitudes towards Continuous Professional Competence and registration

Registration with the Regulator was considered personally important by 89 % (697) of Paramedics/APs and 97 % (381) of EMTs. In addition, 77 % (615) of the Paramedics/APs and 86 % (343) of EMTs stated that CPC was extremely important professionally. Most Paramedic/AP respondents (74 %, 584) agreed that CPC should be a condition of registration to practice, while 95 % (341) of EMTs held that view. 67 % (526) of the Paramedic/AP respondents agreed that Paramedics and APs should maintain evidence of CPC activities to ensure registration, while 82 % (329) of EMTs believed that to be the case.

39 % (307) of Paramedics/APs agreed with the suggestion that those who fail to meet CPC requirements should be allowed to register only at the level below their current registration, but 23 % (179) did not support that proposition.

### CPC activities

**Fig. 3:**
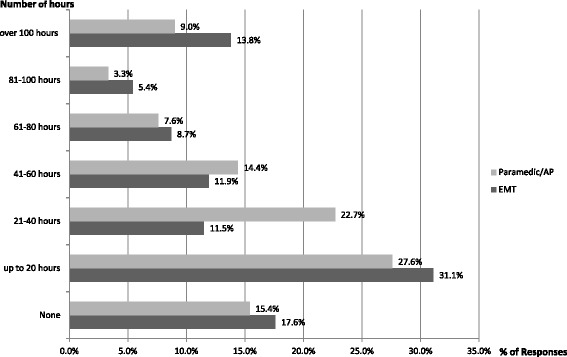
Number of CPC hours recorded in the prior 12-month period by Emergency Medical Technicians (EMTs), Paramedics and Advanced Paramedics (APs)

**Fig. 4:**
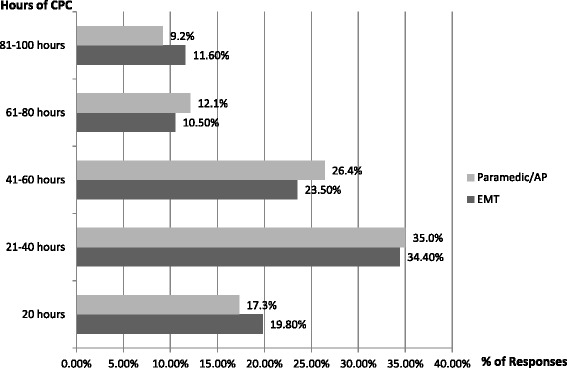
Annual hours of CPC deemed appropriate by Emergency Medical Technicians (EMTs), Paramedics and Advanced Paramedics (APs)

A small majority of Paramedics/APs surveyed (53 %), although not obligated, maintained a professional portfolio at the time of the survey. xThis majority was greater (69 %) in the EMT group. Fifty seven per cent of Paramedics/APs and 51 % of EMTs had completed greater than 20 hours of CPC activities in the prior 12 months, with 20 % of Paramedics/APs and 28 % of EMTs having completed more than 60 hours (Fig. 3). When the Paramedics/APs were queried as to what they believed should be the appropriate levels of CPC required in a 12-month period, 35 % believed 21–40 hours , 26 % believed 41–60 hours, and 17 % believed 20 hours would be adequate (Fig. 4). Yet, 34 % of EMTs believed that 21–40 hours were adequate, 23 % that 41–60 hours, and 20 % (58) that 20 hours would be adequate.

**Fig. 5:**
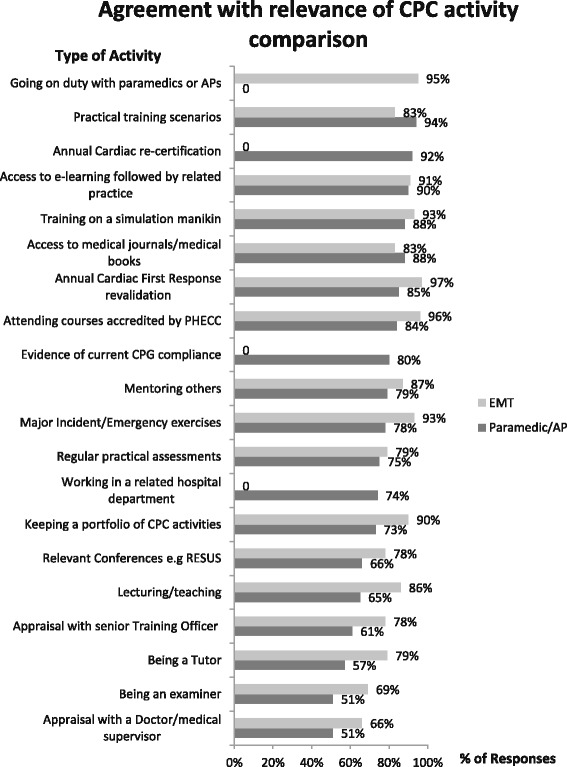
Comparison of activities deemed relevant by Emergency Medical Technicians (EMTs), Paramedics and Advanced Paramedics (APs)

### Consultation regarding specific models of Continuous Professional Competence

Overall, the majority of EMT (88 %) and Paramedics/AP (77 %) respondents favoured the introduction of CPC by the Regulator using a ‘mixed’ model approach of combining ‘mandatory’ and ‘voluntary’ activities, with 84 % of EMTs and 77 % of Paramedics/APs supportive of minimum standard requirements that include evidence of patient care report (PCR) completion, clinical practice guidelines (CPGs) compliance and patient management.

Most respondents considered practical type learning relevant to their roles (Figure 5 and Table 2): practical training scenarios, EMT 83 % (266), Paramedic/Advanced Paramedic 94 % (582); annual cardiac first response revalidation: EMT 97 % (311), Paramedics/APs 85 % (517); access to e-learning followed by related practice EMT 91 % (291), Paramedics/APs 92 % (566); and training on simulation manikins EMT 93 % (297), Paramedics/APs 88 % (535). Ninety five per cent of EMTs (306) surveyed would value the opportunity to complete duties with Paramedics and Advanced Paramedics.

**Table 2:** Relevance of potential CPC activities

Relevant = Very relevant/Relevant Not relevant = Not relevant/Very irrelevant P/AP = Paramedic/Advanced paramedic N/A = Not asked/Not applicable % of total = % of total responses	Relevant responses				Not relevant responses				Total responses for question	
	P/AP	% of total	EMT	% of total	P/AP	% of total	EMT	% of total	P/AP	EMT
Practical training scenarios	582	94 %	266	83 %	9	1 %	2	1 %	613	321
Going on duty with Paramedics or APs	N/A	N/A	306	95 %	N/A	N/A	7	2 %	N/A	321
Annual Cardiac re-certification	566	92 %	N/A	N/A	29	4 %	N/A	N/A	616	N/A
Access to e-learning followed by related practice	555	90 %	291	91 %	11	2 %	5	2 %	617	320
Access to medical journals/medical books	538	88 %	266	83 %	17	3 %	11	3 %	615	320
Training on a simulation manikin	535	88 %	297	93 %	25	4 %	7	2 %	613	321
Attending courses accredited by Regulator	518	84 %	307	96 %	30	5 %	2	1 %	614	319
Annual Cardiac First Response revalidation	517	85 %	311	97 %	60	10 %	6	1 %	611	322
Evidence of current CPG compliance	489	80 %	N/A	N/A	25	4 %	N/A	N/A	611	N/A
Mentoring others	483	79 %	277	87 %	47	8 %	12	4 %	613	317
Major incident/Emergency exercises	480	78 %	297	93 %	34	5 %	7	2 %	612	319
Regular practical assessments	458	75 %	253	79 %	48	8 %	13	4 %	613	319
Working in a related hospital department	453	74 %	N/A	N/A	64	10 %	N/A	N/A	612	N/A
Keeping a portfolio of CPC activities	441	73 %	288	90 %	55	9 %	4	1 %	606	319
Relevant conferences	405	66 %	246	78 %	74	12 %	18	6 %	613	317
Lecturing/teaching	403	65 %	276	86 %	76	12 %	15	5 %	612	319
Appraisal with senior Training Officer (or above)	373	61 %	248	78 %	92	15 %	20	6 %	612	319
Being a Tutor	349	57 %	251	79 %	95	16 %	19	6 %	607	316
Appraisal with a doctor/medical supervisor	309	51 %	207	66 %	115	19 %	37	11 %	610	320
Being an examiner	309	51 %	222	69 %	116	19 %	30	9 %	607	319
Case study review	283	46 %	204	64 %	114	19 %	20	6 %	610	317
Project work	223	37 %	152	48 %	166	27 %	50	16 %	607	318
E-learning modules only and no related practice	203	33 %	109	35 %	210	36 %	101	32 %	607	313
First Aid competitions	N/A	N/A	159	50 %	N/A	N/A	78	25 %	N/A	315
Appraisal of journal publications	188	31 %	124	39 %	147	24 %	62	20 %	607	316

The activities that received the highest ‘not relevant’ responses were: ‘e-learning modules only and no related practice EMT 32 % (101), Paramedics/APs 36 % (210); project work EMT16% (50), Paramedics/APs 27 % (166); appraisal of journal publications EMT 20 % (62), Paramedics/APs 24 % (147); and, for EMTs only, First Aid competitions 25 % (78).

In addition to the practical-type, hands-on activities preferred for CPC maintenance, EMTs, Paramedics and APs also considered the following activities very relevant or relevant in maintaining CPC: access to medical journals/books EMT 83 % (266), Paramedics/APs 88 % (538/615); attending courses accredited by the Regulator EMT 96 % (307), Paramedics/APs 84 % (518/614); evidence of current CPG compliance (EMTs were not asked this question) Paramedics/APs 80 % (489); mentoring others EMT 87 % (277), Paramedics/APs 79 % (483); major incident/emergency exercises EMT 93 % (297), Paramedics/APs 78 % (480/612); regular practical assessments EMT 79 % (253), Paramedics/APs 75 % (458); working in a related hospital department (EMTs were not asked this question) Paramedics/APs 74 % (453); keeping a portfolio of CPC activities EMT 90 % (288), Paramedics/APs 73 % (441); attending relevant conferences EMT 78 % (246), Paramedics/APs 66 % (405); lecturing/teaching EMT 86 % (276), Paramedics/APs 65 % (403); appraisal with a senior Training Officer EMT 78 % (248), Paramedics/APs 61 % (373); being a tutor EMT 79 % (251), Paramedics/APs 57 % (349); appraisal with a doctor/medical supervisor EMT 66 % (207), Paramedics/APs 51 % (309); being an examiner EMT 69 % (222), Paramedics/APs 51 % (309); case study review EMT 64 % (204),Paramedics/APs 46 % (283).

## Discussion

Some literature reports the development of ambulance CPD programmes internationally [7, 9]. Although CPD is more likely to lead to a change in practice when a needs assessment has been conducted [18], literature reporting consultation with practitioners prior to the introduction of such programmes is limited.

This first study of attitudes towards professional competence among EMTs, Paramedics and APs in Ireland suggests a genuine enthusiasm for the introduction of CPC, with all groups indicating that CPC was of personal importance to them. Also there is evidence from their responses that all groups believed in the need for CPC and the link with registration and that the majority of these practitioners were already maintaining CPC portfolios (Fig. 3).

### Demographics

The majority of total responses across all levels were from males 941 (80 %) with females at 234 (20 %) (Table 1) and from registrants within the National Ambulance Service (NAS). This is unsurprising as the ambulance services in Ireland are provided, predominately, by the National Ambulance Service (NAS). In addition to the NAS, Dublin Fire Brigade provides ambulance services through twelve ambulance vehicles based throughout Dublin City. Both services are male dominated and employ Paramedics and Advanced Paramedics with few EMTs in either service. The NAS has, since 2012, commenced recruiting EMTs into the ambulance service but these are few in number currently standing at approximately one hundred (i.e., circa 7 % of the workforce). Therefore, most registered EMTs are members of the Voluntary Organisations. While it could be argued these EMTs are not full-time professionals, they are committed volunteers on a national regulatory and professional register and do perform duties at large events.

### CPC activities

This study identified a number of useful topics and activities that could be considered for the purpose of CPC, and has identified some areas of low CPC priority for registrants.

Specifically, practical hands-on training using simulation manikins, team-based activities or e-learning followed by practical skills were preferred over non-practical/theory-type activities. Also, there were less negative responses regarding activities related to practical skills than to theoretical skills. A study with Irish APs reinforced the concept of practical-type learning as a preferred methodology and as an effective way of maintaining competence [6] and indeed scenario-based simulations have been used since 2007 as part of routine continuing education programmes by some American emergency medical services [9]. Interactive methods, for the purposes of CPD, such as team-based learning and case-based learning, as compared to lectures, impart sustainable knowledge and lead to high satisfaction among participants [19]. For example, Davis et al. [20] in their systematic review found that interactive and mixed educational sessions were associated with a significant effect on physicians’ performance, effected change in professional practice and, on occasion, healthcare outcomes.

The least relevant activities, identified by both the EMT and Paramedic/AP groups were associated with non-skills/practical, individually-based, passive activities: e-learning modules only and no related practice.

Other activities regarded as less relevant were: project work and appraisal of journal publications. This is quite different to results seen from other professions who have tended to prefer attending conferences, lectures and reading of relevant journals [14, 21] even though there is little evidence to suggest that attending conferences had any direct impact on improving professional practice [22].

Studies on cardiac nurses and dietitians [14, 23] have shown that journal reading was a popular preference. However, for doctors the effectiveness of continuous medical education (CME) increases as the intervention strategy becomes more active, while activities classed as passive are associated less with changes in physician performance or patient outcome [22].

### Model of CPC

Groups were split in relation to opinion on annual hours of CPC that should be required; both cohorts responded similarly, and with the highest response rate to this section, stating that 21–40 hours were adequate (see Fig. 4). The majority of both cohorts surveyed favoured a ‘mixed’ model approach for CPC with a similar number supporting the idea of minimum standard requirements which involved evidence of patient care. This ‘mixed’ model approach would allow for a ‘compulsory’ element to the CPC requirements and an additional ‘voluntary’ allowance that is still required but would allow the registrant some flexibility in deciding which activities to choose.

The benefit of mandatory CPD in healthcare professions has been debated. O’Connor’s [24] study on motivating factors for nurses participating in continuing education (CPD) suggested that the mandatory nature of the education had little influence in motivating participation, while Lee et al. [25] found that 66 % of Australian radiographers thought CPD should be voluntary. Friedman and Woodhead [26] suggested that those professional bodies utilising compulsory or mixed policies with respect to CPD were likely to be promoting CPD as a means of maintaining competence.

Regarding sanctions, the majority of EMTs and Paramedics/APs agreed that the practitioner should not be allowed to re-register at their current level if they failed to meet the CPC requirements. This finding is higher than from some other healthcare professions, for example 42 % of pharmacists surveyed [27] favoured sanctions yet few dietitians favoured disciplinary action for those who failed to meet the registration requirements [23].

### Limitations

While this first study of attitudes towards CPC among EMTs, Paramedics and APs in Ireland involved a national sample, we acknowledge some methodological considerations may limit generalisability. We report data from 1188 responses which is a relatively large number that compares well with other reported surveys [28–31], representing 43 % of all registered EMTs and 43 % of all registered Paramedics and Advanced Paramedics. In addition, it is important to note that the response rate from the Advanced Paramedic cohort represented 82 % of those registered. Our study was limited to those with valid email addresses and clearly those for whom the subject area was of interest responded. Further research following the introduction of CPC for all three levels of Irish registered pre-hospital practitioners, may expand upon these findings.

## Conclusions

There is a paucity of research conducted with registered pre-hospital practitioners in Ireland. This survey is the first to ascertain the opinions of EMTs, Paramedics and APs regarding CPC. While there is evidence of the need for pre-hospital practitioners maintaining competence in other ambulance services internationally e.g., Australia, UK and Canada, their guidelines are less prescriptive. In the UK, for example, the Health and Care Professions Council that regulates Paramedics, state in its guidance document that ‘there is no automatic link between [evidence of] CPD and your competence’ as this is not directly linked to the legislation which established that body (the Health and Social Work Professions Order 2001). Also, although all their registrants must maintain ‘a continuous up-to-date and accurate record of their CPD activities’, this requirement is linked to a set of professional competencies associated with the profession and are less specific. This too is the case in Australia [32] and Canada where they use their standards of competence to encourage CPD in a non-specific and generic manner across practitioner levels: ‘Participate in continuing education and professional development; develop personal plans for continuing professional development; describe common quality assurance and enhancement processes’ [33].

This study further suggests that there is willingness on behalf of Irish EMTs, Paramedics and APs to engage with CPC, which is viewed as extremely important. Respondents considered it appropriate to link CPC with registration to practice and that there should be sanctions against those who do not meet CPC requirements.

The results of this survey demonstrate, at the very least, that emphasis will need to be placed on availability/provision of a compulsory ‘mixed’ model approach of CPC. This mixed model approach should include evidence of patient contact. Indeed, this varied model of CPC is also encouraged by the UK Health and Care Professions Regulator, requiring registrants to ‘demonstrate that their activities are a mixture of learning activities relevant to current or future practice’ and, although there is no explicit link to patient contacts guidelines suggest the use of critical reviews and case studies that could imply patient contacts [34].

The mixed model of CPC includes activities that are practically orientated: practical training scenarios; annual cardiac recertification; e-learning followed by related practice; training on simulation manikins. Conversely, there is less interest in non-skills/practical, individual passive learning activities: e-learning alone and no related practice; project work, journal reviews. Somewhere between twenty to sixty hours of CPC activities per annum would appear to be acceptable to Irish practitioners.

## Additional file

Additional file 1:
**Core Questionnaire Summary.** (DOCX 25 kb)
