# Supplementary material for: A national study of Continuous Professional Competence (CPC) amongst pre-hospital practitioners
Source: BMC Health Serv Res. 2015 Dec 2;15:532. doi: 10.1186/s12913-015-1197-1 (PMC4668695; doi:10.1186/s12913-015-1197-1)
Supplement: Additional file 1: — Core Questionnaire Summary. (DOCX 25 kb) [file 12913_2015_1197_MOESM1_ESM.docx]

**Core Questionnaire Summary**

### Gender and registration level

Gender

- Male
- Female

**Registration Status with Regulatory Body (PHECC)**

- Advanced Paramedic
- Advanced Paramedic Intern
- Advanced Paramedic Trainee
- Paramedic
- Paramedic Intern
- Paramedic Trainee
- Emergency Medical Technician (EMT)

### Total number of responses from EMTs, paramedics and advanced paramedics based on organisation (Demographics)

- Permanent Defence Forces
- Private Ambulance Services
- Dublin Fire Brigade
- HSE – National Ambulance Service
- Civil Defence
- Order of Malta
- St. John Ambulance Brigade
- Irish Red Cross
- Irish Coastguard
- An Garda Siochana (Police)
- Army Reserve
- Fire Service
- Other

### *Attitudes towards Continuous Professional Competence and Registration*

Registration with the national Regulator was considered personally important

- By paramedics/advanced paramedics
- EMTs
- Options

Agree

Disagree

**Do you consider CPC extremely important?**

- By Paramedics/Aps
- EMTs
- Options

Agree

Disagree

**Should CPC be a condition of registration to practice?**

- By Paramedics/Aps
- EMTs
- Options

Agree

Disagree

**Do you think paramedics/advanced paramedics/EMTs should maintain evidence of CPC activities to ensure registration?**

- Options

Agree

Disagree

**Should those who fail to meet the CPC requirements be allowed to register at the level below their current registration?**

- paramedics/advanced paramedics
- EMTs
- Options

Agree

Disagree

**Do you maintain a professional portfolio at the time of this survey?**

- paramedics/advanced paramedics
- EMTs
- Options

Agree

Disagree

**What do you believe should be the appropriate levels of CPC required in a 12-month period?**

- paramedics/advanced paramedics
- EMTs
- Options

None

Up to 20 hours

21-40 hours

41-60 hours

61-80 hours

81-100 hours

Over 100 hours

### Number of CPC hours recorded in the previous 12-month period by EMTs, paramedics and advanced paramedics?

- Options

None

Up to 20 hours

21-40 hours

41-60 hours

61-80 hours

81-100 hours

Over 100 hours

### Number of annual hours of CPC deemed appropriate by EMTs, paramedics and advanced paramedics

- Options

20 hours

21-40 hours

41-60 hours

61-80 hours

81-100 hours

### Consultation regarding specific models of Continuous Professional Competence

**Would you favour the following method?:**

**The introduction of CPC by the regulatory body using a ‘mixed’ model approach of combining ‘mandatory’ and ‘voluntary’ activities**

- paramedics/advanced paramedics
- EMTs

**Would you favour the following method?:**

**Minimum standard requirements that include evidence of Patient Care Report (PCR) completion, clinical practice guidelines (CPGs) compliance and patient management**

- paramedics/advanced paramedics
- EMTs

**Is practical type learning relevant to your role?**

Yes / No

**Relevance of potential CPC activities**

Do you think the following are relevant or not relevant potential CPC activities?

- Practical training scenarios
- Going on duty with paramedics or advanced paramedics
- Annual cardiac re-certification
- Access to e-learning followed by related practice
- Access to medical journals/medical books
- Training on simulation manikins
- Attending courses accredited by PHECC
- Annual cardiac First Response revalidation
- Evidence of current CPG compliance
- Mentoring others
- Major Incident/Emergency exercises
- Regular practical assessments
- Working in a related hospital department
- Keeping a portfolio of CPC activities
- Relevant conferences
- Lecturing/teaching
- Appraisal with senior Training Officer (or above)
- Being a Tutor
- Appraisal with a doctor/medical supervisor
- Being an examiner
- Case study review
- e-learning modules only and no related practice
- Project work
- e-learning modules only and no related practice
- First Aid competitions
- Appraisal of journal publications

**Question for EMTS:**

**Would you value the opportunity to complete duties with paramedics and advanced paramedics?**

Yes / No

**Hands-on activities preferred for CPC maintenance (EMTs, paramedics and advanced paramedics)**

**Do you think the following are relevant or not relevant for CPC maintenance?**

- Access to medical journals/books
- attending courses accredited by the Regulator
- evidence of current CPG compliance (EMTs were not asked this question)
- mentoring others (EMTs were not asked this question)
- major incident/emergency exercises
- regular practical assessments
- working in a related hospital department (EMTs were not asked this question)
- keeping a portfolio of CPC activities
- attending relevant conferences
- lecturing/teaching
- appraisal with a senior Training Officer
- being a tutor
- appraisal with a doctor/medical supervisor
- being an examiner
- case study review

**Is CPC of personal importance to you?**

Yes / No

**Do you believe in the need for CPC and the link with registration?**

Yes / No

**Do you maintain a CPC portfolio?**

Yes / No

**Do you believe that the practitioner should not be allowed to re-register at their current level if they failed to meet the CPC requirements?**

Yes / No
